# Influence of the coronavirus disease 2019 pandemic on the post-graduate career paths of medical students: a cross-sectional study

**DOI:** 10.1186/s12909-023-05021-6

**Published:** 2024-01-10

**Authors:** Ayumu Nishimura, Tomoko Miyoshi, Fumio Otsuka, Akihiro Matsukawa

**Affiliations:** 1 Okayama University Medical School Faculty of Medicine, Okayama, Japan; 2 Department of General Medicine, Graduate School of Medicine, Dentistry and Pharmaceutical Sciences, Okayama University, Okayama, Japan; 3 Department of Pathology and Experimental Medicine, Graduate School of Medicine, Dentistry and Pharmaceutical Sciences, Okayama University, Okayama, Japan

**Keywords:** COVID-19 pandemic, Medical students, Career path, Training hospitals

## Abstract

**Background:**

The World Health Organization first declared the coronavirus disease 2019 (COVID-19) pandemic in March 2020 and announced the end of the emergency in May 2023. Additionally, the COVID-19 pandemic significantly impacted individuals globally, including medical students. Although the COVID-19 pandemic increased online education, it restricted clinical training, extracurricular activities, and interprovincial travel. Therefore, this study aimed to examine whether the COVID-19 pandemic influenced the choice of training hospitals and career paths among 3rd- to 6th-year medical students in Japan.

**Methods:**

We developed a questionnaire comprising 21 multiple-choice and 1 open-ended questions, which was administered anonymously via online platforms. The survey targeted Japanese medical students to obtain insights into their preferences for training hospitals and career paths during the COVID-19 pandemic. Participants included 4th- to 6th-year medical students from 51 medical schools in Japan. The survey was conducted through student networks from 8 February 2022 to 20 March 2022.

**Results:**

Overall, 507 medical students participated in the survey, with representation from various academic years as follows: 102 (20.1%), 134 (26.4%), 121 (23.9%), and 150 (29.6%) students from the 3rd, 4th, 5th, and 6th year, respectively. Of these, 338 (66.6%) students reported that the COVID-19 pandemic had influenced their choice of training hospitals. The degree of the influence varied based on the university region and the student year. However, most of the students (473, 93.3%) did not change their course for clinical, basic research, or administrative pathways due to the COVID-19 pandemic. Among the clinically oriented students, 391 (77.2%) did not change their preferred speciality.

**Conclusions:**

The COVID-19 pandemic influenced medical students’ choice of training hospitals. Although many students believed that the pandemic would not change their career choices, our results indicate a potential subconscious trend to avoid internal medicine, which is the speciality most directly involved in treating patients with COVID-19.

**Supplementary Information:**

The online version contains supplementary material available at 10.1186/s12909-023-05021-6.

## Background

The World Health Organization first declared the coronavirus disease 2019 (COVID-19) pandemic in March 2020 and subsequently announced the end of the emergency in May 2023 with the implementation of appropriate infection control measures, the development of vaccines and diagnostic technical tools, and the mutation of the coronavirus. The COVID-19 pandemic, which spanned 3 years and 3 months, enormously affected people worldwide, including medical students.

Consequently, medical education shifted from in-person to online education, and clinical practice was suspended in approximately 90% of medical schools worldwide due to many social constraints [1, 2]. A study by Shahrvini et al. showed that distance learning provided students with the flexibility to learn at their own pace; however, 43.3% of students felt unprepared to begin clinical practice [3]. Students felt insecure about their ability to participate and whether they had acquired the appropriate clinical skills due to insufficient hands-on learning [3]. The COVID-19 pandemic also influenced the formation of medical students’ identity as physicians. Insufficient on-campus learning, limited peer interaction, reduced direct involvement in patient care, and increasing barriers to professional identity formation pose significant challenges for medical students in finding their place in the medical field and developing confidence in their value as healthcare professionals [4]. Deng et al. reported that approximately 10% of medical students experienced an increase or decrease in their awareness of becoming a physician during the COVID-19 pandemic [5]. Byrnes et al. reported that the COVID-19 pandemic influenced the speciality choices of approximately 20% of medical students in the United States of America [6]. Medical students could not explore specialities of interest, strengthen their residency applications with letters of recommendation, participate in away rotations, or opt for an additional year of medical school [6]. In addition, Elsawy et al. suggested that the lack of elective practice reduced the opportunities for students to explore career paths and put them at a disadvantage when applying to speciality programs [7]. Consequently, reduced clinical practice may result in decreased exposure to role models [8]. As mentioned above, the COVID-19 pandemic had a significant impact on medical students. However, no reports exist on post-graduate career path surveys in Japan, where the physician training system differs from that in the United States of America.

In Japan, students enrol in a 6-year medical school program after high school graduation. Subsequently, after graduating from medical school, individuals aspiring to become clinicians must pass the national medical examination for medical practitioners and undergo an additional 2 years of initial clinical training [9]. Following this, many physicians opt to pursue specialised programs [10, 11]. Therefore, this study aimed to examine whether the COVID-19 pandemic influenced the choice of training hospitals and career paths of Japanese medical students.

## Methods

### Study design

We conducted a cross-sectional, anonymous, online survey that targets medical students across Japan during the COVID-19 pandemic, spanning from 8 February 2022 to 20 March 2022. The questionnaire used was adapted from the study conducted by Byrne et al., [6] with modifications to align with the Japanese medical school system. We added questions about the university and family home areas, the hospital area for clinical training, and the choice of training hospital (Q3–Q7). The questionnaire comprised 21 multiple-choice questions and a free-text response (See Supplementary Table 1, Additional File 1). Specifically, the survey was initially distributed online to 3rd- to 6th-year medical students at 82 medical schools in Japan, and responses were received from students at 51 schools. During the survey, the 6th-year medical students had already undergone the national examination for medical practitioners and completed the training hospital matching process; the 5th-year medical students had completed their core rotation and partly commenced their elective rotation; the 4th-year medical students had partly completed their core rotation; and the 3rd^−^year medical students were attending clinical medicine lectures in preparation for clinical practice. The first author, AN, was a 5th-year medical student at the time of the study and distributed the questionnaire to 3rd- to 6th-year medical students through grade representatives at Okayama University. Regarding the other medical schools, peers with social relations, including club activities, were asked to complete the questionnaire, and they distributed the questionnaire through their respective grade class contact network. The questionnaires were distributed in the appropriate academic year following the researcher’s explanation of the study. Furthermore, participation in the survey was voluntary. The study was reviewed and approved by the Okayama University Ethics Committee (approval number: 2208-006), and its protocols were performed following the relevant Strengthening the Reporting of Observational Studies in Epidemiology guidelines and regulations. Informed consent was obtained by checking the consent confirmation box on the unmarked questionnaire included in the preface of the survey e-mails from all participants.

### Statistical analysis

The differences in student responses by region were compared between the Kanto region, where Tokyo is located, and other regions. Fisher’s test was conducted using the Bell Curve for Excel statistics (Social Survey Research Information Co., Ltd., Tokyo, Japan). All other comparison tests were analysed using EZR version 1.55 (Saitama Medical Centre, Jichi Medical University, Saitama, Japan), and the Bonferroni multiple comparison tests were applied. Statistical significance was set at *P* < 0.05.

## Results

### Characteristics of the enroled students

**Fig. 1:**
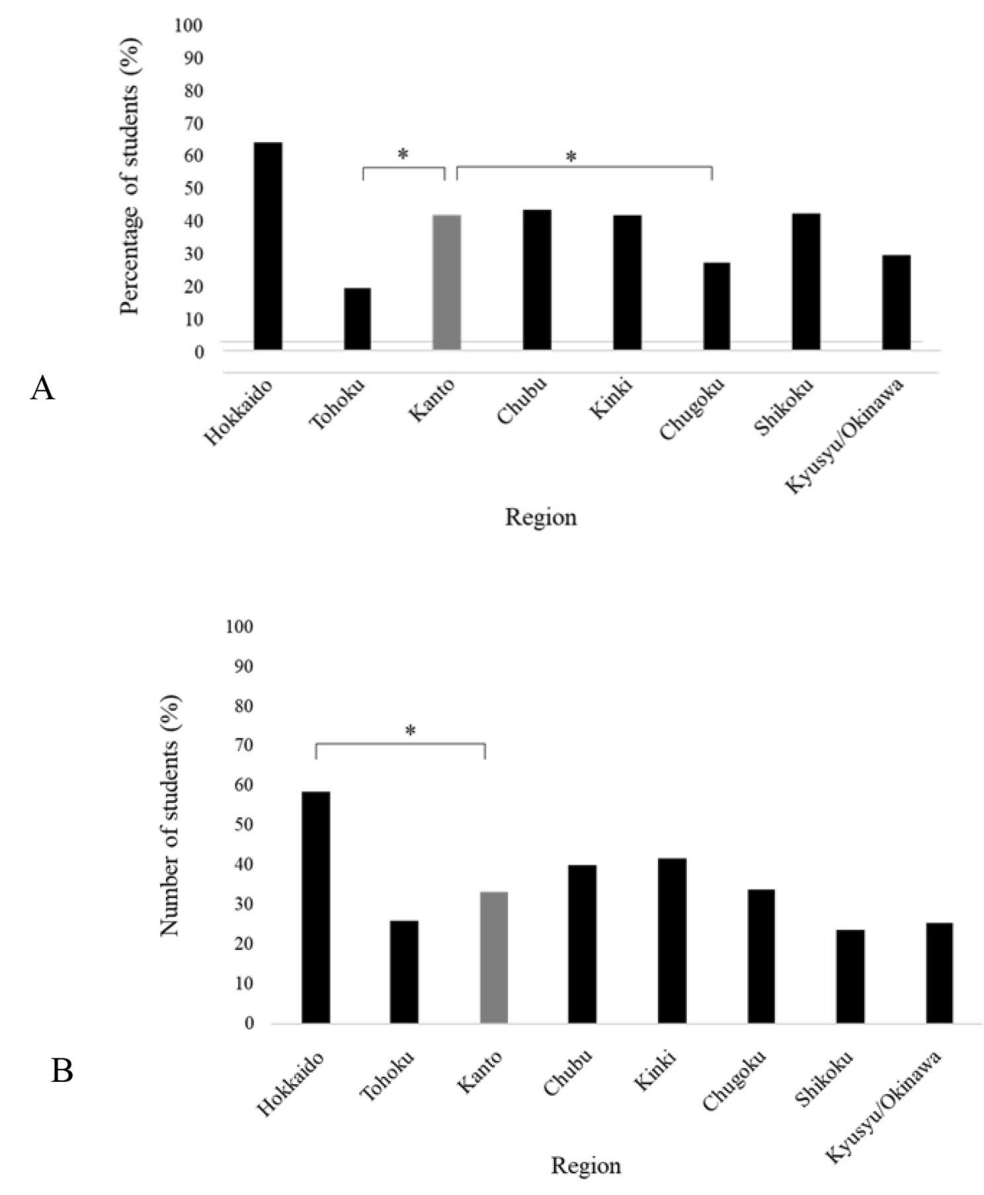
Characteristics of medical students who responded by prefecture where the university is located. (**a**) Percentage of medical students by region whose parents’ home and university are in the same prefecture. Comparison based on the Kanto region, the capital of Japan. (**b**) Percentage of medical students by region whose university and desired training hospital are located in the same prefecture. Comparison based on the Kanto region, the capital of Japan

The responses from 507 medical students were analysed. Among these, 102 (20.1%), 134 (26.4%), 121 (23.9%), and 150 (29.6%) medical students were in their 3rd, 4th, 5th, and 6th years, respectively (Table 1). In total, 22 (4.3%), 27 (5.3%), 80 (15.8%), 42 (8.3%), 72 (14.2%), 112 (22.1%), 50 (9.9%), and 102 (20.1%) participants were in the Hokkaido, Tohoku, Kanto, Chubu, Kinki, Chugoku, Shikoku, and Kyushu and Okinawa regions, respectively (Table 1). Among the medical students who reported that their university and hometown were in the same prefecture, 14 (63.6%), 5 (18.5%), 33 (41.3%), 18 (42.9%), 30 (41.7%), 30 (26.8%), 21 (42.0%), and 30 (29.4%) were in the Hokkaido, Tohoku, Kanto, Chubu, Kinki, Chugoku, Shikoku, and Kyushu/Okinawa regions, respectively. The Tohoku and Chugoku regions had significantly more medical students from different prefectures than the Kanto region, where Tokyo is located (Fig. 1A).

**Table 1:** Characteristics of the medical students who responded

Regions	N (%)	3rd-year MS	4th-year MS	5th-year MS	6th-year MS
Hokkaido	22 (4.3)	4	6	8	4
Tohoku	27 (5.3)	7	13	4	3
Kanto	80 (15.8)	15	18	24	23
Chubu	42 (8.3)	17	8	11	6
Kinki	72 (14.2)	12	17	12	31
Chugoku	112 (22.1)	13	41	28	30
Shikoku	50 (9.9)	6	7	11	26
Kyushu/Okinawa	102 (20.1)	28	24	23	27
Total number (%)	507 (100)	102 (20.1)	134 (26.4)	121 (23.9)	150 (29.6)

Abbreviations: MS, Medical student

### Preferences for training hospitals by region

Medical students who indicated that their university and preferred training hospital were located in the same prefecture were 13 (59.1%), 7 (25.9%), 27 (33.8%), 17 (40.5%), 30 (41.7%), 38 (33.9%), 12 (24.0%), and 26 (25.5%) in the Hokkaido, Tohoku, Kanto, Chubu, Kinki, Chugoku, Shikoku, and Kyushu/Okinawa regions, respectively. More medical students studying in the Hokkaido region preferred clinical training in the prefecture where their university is located than those studying in the Kanto region (Fig. 1B). Notably, the percentage of medical students in other regions was not significantly different from that in the Kanto region.

The common reason regarding the choice of training hospital (multiple choice) among the 150 (29.6%) 6th-year medical students was that the program appeared interesting regardless of the location, with 45 (30.0%). This was followed by 41 (27.3%) medical students who reported that their choice was because of the proximity to their hometown, 34 (22.7%) because of their university-affiliated hospital, 28 (18.7%) because there was a medical school they wanted to join in the future, 7 (4.7%) because they could not go outside the prefecture during the pandemic, and 18 (12.0%) for other reasons (such as family, regional quota scholarship, and university mission) (Table 2). For the 3rd- to 5th-year medical students, 157 (44.0%) mentioned that proximity to the hometown was the most common reason, followed by 89 (24.9%) who found the program interesting regardless of location (Table 2).

**Table 2:** The reasons the university and the desired training hospital for students, which are in the same prefecture under the COVID-19 pandemic, influenced the choice of training hospital

The reasons the university and the desired training hospital for students are in the same prefecture	3rd- to 5th-year MS	6th-year MS
	N (%)	N (%)
Close to my parents’ home.	157 (44.0)	41 (27.3)
There is a hospital in the prefecture where the program I am interested in is located.	89 (24.9)	45 (30.0)
Affiliated hospital of my university.	68 (19.0)	34 22.7)
There is a medical department that I want to join in the future.	71 (19.9)	28 (18.7)
I do not know because I have not started thinking about it yet.	41 (11.5)	0 (0.0)
Regional quota.	11 (3.1)	4 (2.7)
Could not go outside the prefecture where my university is located due to the COVID-19 pandemic.	8 (2.2)	7 (4.7)
Others (family, regional scholarships, and university mission).		18 (12.0)

Abbreviations: COVID-19, coronavirus disease 2019; MS, Medical student; N, Number

### Influence of the COVID-19 pandemic on the choice of residency training hospital

**Fig. 2:**
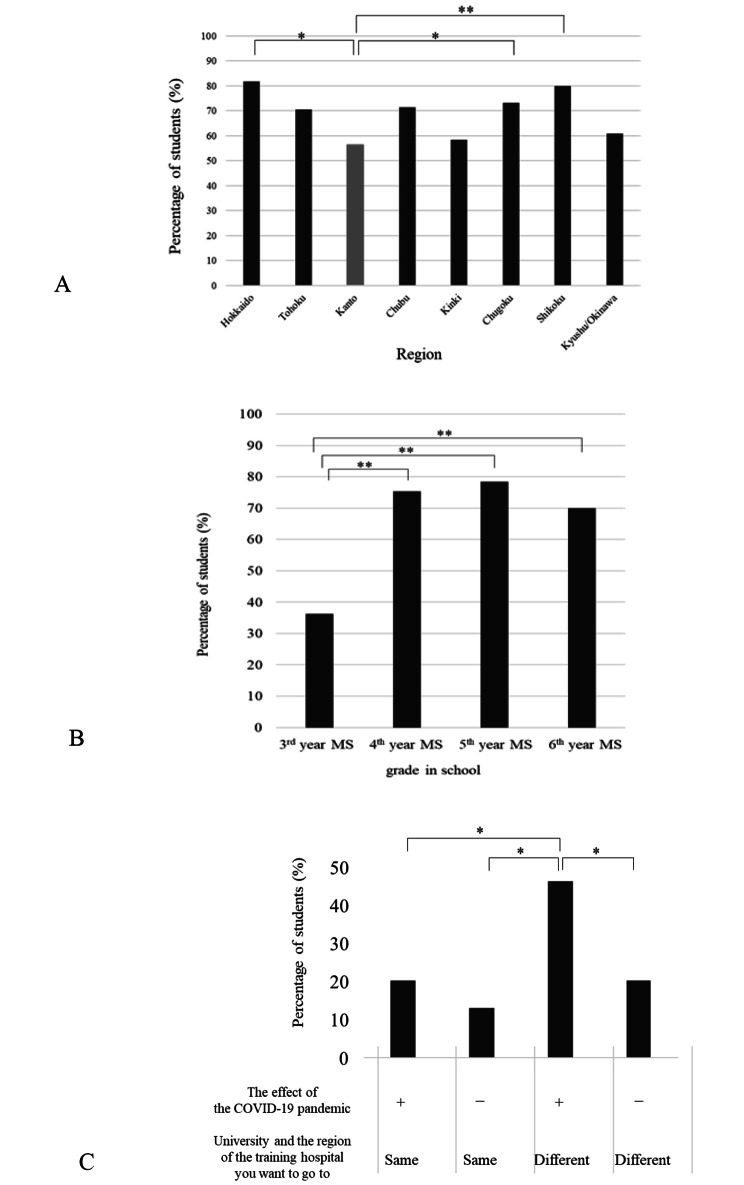
Influence of the coronavirus disease 2019 (COVID-19) pandemic on the choice of residency training hospital. (**a**) Percentage of medical students by region where the COVID-19 pandemic influenced their choice of training hospital. Comparison based on the Kanto region, the capital of Japan. (**b**) Percentage of medical students per grade level for whom the COVID-19 pandemic influenced their choice of training hospital. (**c**) Percentage of medical students who were influenced in their choice of training hospital by the COVID-19 pandemic and the prefecture where the training hospital is located. *p < 0.05, **p < 0.01, Abbreviations: MS Medical Student; COVID-19, coronavirus disease 2019

Overall, 338 (66.6%) medical students responded ‘yes’ when asked whether COVID-19 influenced their choice of residency training hospital. In contrast, when analysed by regions, more medical students in the Hokkaido, Chugoku, and Shikoku regions responded that COVID-19 remarkably influenced their choice of training hospitals than those in the Kanto region (Fig. 2A). All medical students in the 4th-, 5th- and 6th- year were equally influenced by the pandemic (75.4%, 78.7%, and 70.0%, respectively), whereas the 3rd-year medical students were significantly less influenced (36.6%) (Fig. 2B). Medical students whose training hospitals were in a prefecture different from their university were most influenced by their choice of training hospital (Fig. 2C).

Among the medical students who responded that their choice of training hospital was influenced, 335 (99.1%) and 150 (44.3%) reported a negative and positive influence, respectively. Reasons for negative influence included the following: 246 (73.4%) medical students had difficulty making an on-site visit; 220 (65.7%) felt that online information sessions did not provide sufficient information about the program/hospital/staff; 55 (16.4%) had limited opportunity to practice interviewing and matching tests with classmates; 15 (4.5%) reported that travel restrictions made them more competitive in the prefecture where the university is located (6th-year medical students only); 15 (4.5%) experienced limitations in participating in away rotations; and 10 (3.0%) had difficulty in preparing for the final online interview (6th-year medical students only) (Table 3, upper). Furthermore, positive reasons included more time to gather information and consider hospitals (87 responses, 58.0%), online information sessions were easier to participate in, and students had more opportunities to appeal to staff (24 responses, 16.0%) (Table 3, lower).

**Table 3:** The reasons the COVID-19 pandemic influenced the choice of training hospitals

	N (%)
**Negative reasons (Multiple answers available)**	
Difficult to go on an on-site visit	246 (73.4)
Online information sessions are insufficient in conveying the atmosphere of the program/hospital/staff	220 (65.7)
Limited opportunity to practice for the interview and matching examination with classmates	55 (16.4)
The travel restrictions made it more competitive within the prefecture where the university is located. (6th-year medical students only)	15 (4.5)
Participation in away rotations is limited	15 (4.5)
Difficult to prepare for the final interview online (6th-year medical students only)	10 (3.0)
**Positive reasons (Multiple answers available)**	
More time to gather information and consider hospitals that were not on my list of choices	87 (58.0)
Online information sessions gave more opportunities to appeal to the program staff. (Beneficial for programs that are far away and would have allowed a visit during vacation periods)	24 (16.0)

Abbreviations: COVID-19, coronavirus disease 2019; MS, Medical student; N, Number

### Influence of the COVID-19 pandemic on professional interests and future career

**Fig. 3:**
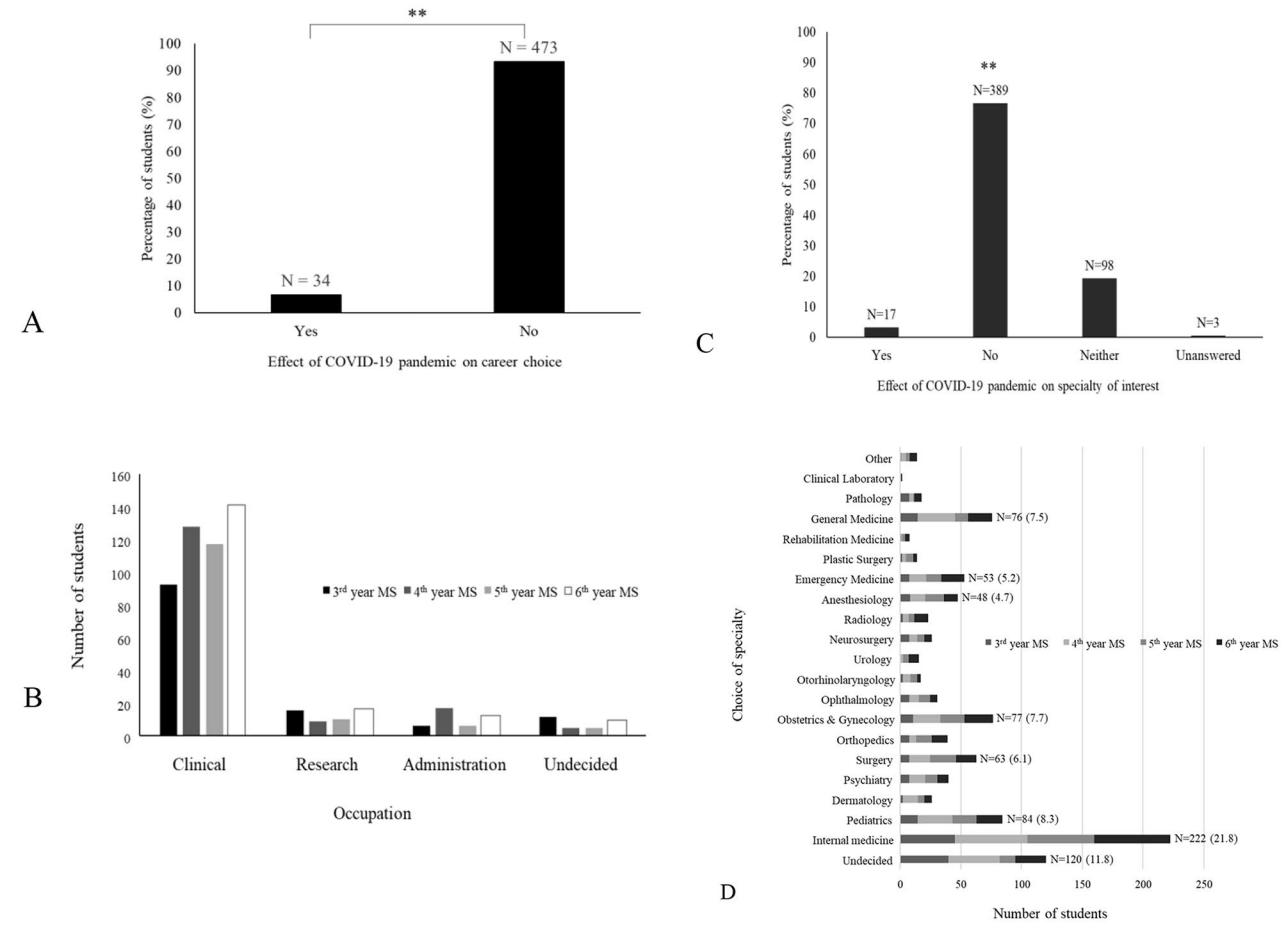
Influence of the coronavirus disease 2019 (COVID-19) pandemic on professional interests and future career. (**a**) Number of medical students for whom the COVID-19 pandemic influenced their career choice. The number above the bar is the number of students. (**b**) The career medical students aspired to pursue in the future by grade level. (**c**) Percentage of medical students whose choice of speciality of interest was influenced by the COVID-19 pandemic. The number above the bar is the number of students. (**d**) Number of medical students interested with multiple choices of speciality by grade level

Regarding the influence of the COVID-19 pandemic on their career paths, only 34 (6.7%) of the total medical students responded ‘yes’ (Fig. 3A). For most medical students, there was no change in their career aspirations. Regarding their future career aspirations, 477 (94.1%) medical students aspired to become physicians, 50 (9.9%) basic researchers, 41 (8.1%) managers, and 30 (5.9%) were undecided (Fig. 3B). Only 17 (3.4%) medical students made changes to their intended career paths (Fig. 3C). Notably, two medical students stated in their free-text responses that they have become more interested in public health. Regarding the specialities that the medical students aspired to pursue in the future (multiple responses), internal medicine was the most preferred speciality for 222 (21.8%) medical students, followed by paediatrics for 84 (8.3%), obstetrics and gynaecology for 77 (7.7%), general practice for 76 (7.5%), surgery for 63 (6.1%), emergency medicine for 53 (5.2%), and anaesthesiology for 48 (4.7%) (Fig. 3D).

## Discussion

This study aimed to clarify the influence of the COVID-19 pandemic on the choice of training hospitals and career paths among 3rd- to 6th-year medical students in Japan. The online questionnaire revealed that the COVID-19 pandemic more likely influenced medical students from the Hokkaido, Chugoku, and Shikoku regions in their choice of training hospital than those from the Kanto region, where the capital Tokyo is located. Notably, medical students whose home university and preferred training hospitals were in different prefectures were more influenced by the COVID-19 pandemic. The reasons included ‘difficulty to go on an on-site visit’ and ‘online information sessions are insufficient in conveying the atmosphere of the program/hospital/staff’. However, some medical students also mentioned positive reasons, such as ‘more time to gather information and consider hospitals that were not on their list of choices’. Specifically, most medical students indicated that the pandemic did not change their professional interests or specialities, and the most commonly preferred speciality was internal medicine, followed by paediatrics, obstetrics and gynaecology, and general practice.

This study’s findings highlight regional differences in the consequence of the COVID-19 pandemic on the selection of training hospitals. These differences may be attributed to two potential factors. First, there are constraints on mobility imposed by the COVID-19 pandemic. In Japan, a series of four emergency declarations were issued between April 2020 and September 2021, restricting movement across prefectures. Particularly, the Chugoku and Shikoku regions have small populations, with only 6.8% and 4.1% of the total number of training hospitals in the region, respectively. Therefore, medical students were largely restricted from travelling from one prefecture where their university was situated to another, potentially influencing their choice of training hospital since they could not physically visit these institutions. Second, educational inequalities may play a role. Although educational inequality is considered to be lower in Japan than in the United States of America [12], they still exist [13]. Particularly, the income of the parents of medical students in urban regions is high [13]. Therefore, urban medical students may have access to a well-rounded education and enrol in rural medical colleges. In this study, more medical students from the Tohoku and Chugoku regions were from outside the prefectures than those from the Kanto region.

However, the consequence of the COVID-19 pandemic on the choice of training hospital differed between the grades. Sixth-year medical students who had already chosen a training hospital were more focused on their training programme and future career path, which likely influenced their choice of training hospital. In Japan, clinical training begins in the 4th year, and medical students usually visit training hospitals. In this study, 4th- to 6th-year medical students were also influenced in their choice of training hospitals by the COVID-19 pandemic; however, most of the medical students reported that their career paths did not change. According to the 2018 survey by the Japanese Ministry of Health, Labour, and Welfare, 96.3% of doctors were employed as clinicians, 1.6% as researchers, and 1.3% were engaged in governmental and other organisational roles [14]. This survey’s results also mirrored the inclination of many medical students to pursue careers as clinicians.

Interestingly, only 6.7% of Japanese medical students had their choice of future speciality affected, even during the COVID-19 pandemic, whereas approximately 20% of medical students in the United States of America experienced a similar impact, as revealed in a comparable survey [6]. The difference in post-medical school trajectories could be a contributing factor, with medical students in the United States of America transitioning to department-specific residency programs after 1 year of internship, whereas Japanese medical students undergo a common 2-year initial training program before entering a department-specific specialist program. Studies have also shown that exposure to various specialities is essential for the professional development of medical students and is an important step in recognising possible career choices [6, 8]. Nara et al. reported that 56 of the 74 medical schools in Japan offer rotations in all departments during clinical training, which may have been based on the premise of allowing medical students to gain experience in many departments during clinical training [15].

The results of the survey on medical students’ future professional interests (multiple responses) revealed that the most preferred career path was internal medicine, followed by paediatrics, obstetrics and gynaecology, general practice, surgery, emergency medicine, anaesthesiology, and psychiatry. The combined number of applicants for internal and general medicine in this study was 29.3%. Compared with a pre-pandemic survey of career paths in Japan [16], where 37.1%, 11.1%, 8.2%, 5.9%, 3.3%, 3.1%, and 2.7% of the medical students preferred internal medicine (including infectious diseases and psychosomatic medicine), surgery, paediatrics, obstetrics and gynaecology, psychiatry, anaesthesiology, and emergency medicine, respectively, the COVID-19 pandemic appears to have significantly reduced the number of applicants for internal medicine. Several reports exist on burnout among medical doctors and physician trainees involved in COVID-19 treatment [17, 18], and medical students may have unconsciously avoided the medical field (i.e., internal medicine and general medicine) that deals with infectious diseases in the context of the current COVID-19 pandemic.

Regarding the reasons that influenced the choice of professional interests, 34 medical students stated that the COVID-19 pandemic positively influenced their choice of career path. Two medical students expressed developing an interest in public health, and one mentioned becoming interested in psychiatry after witnessing people around them experiencing mental health issues. However, there was also a potentially negative effect of the COVID-19 pandemic, as one medical student failed to recognize the benefits of clinical practice.

This study had some limitations. First, only 51 of the 82 universities participated in the survey, with 507 questionnaires completed, representing only 1.4% of all medical students in the country. Additionally, the relatively limited sample size, the higher number of responses from the Chugoku region, where the researchers belong, and the students’ cooperation in the survey suggest a potential bias toward speciality departments. Second, medical students in this study were allowed to select more than one speciality of their choice, making direct comparisons with previous surveys challenging. Third, Japan has regional quota students who undergo training in a specific region [13]; however, this study did not investigate the presence of regional quota students, and the consequence of regional quota students on the choice of training hospitals remains unknown.

## Conclusions

The COVID-19 pandemic influenced medical students’ choice of training hospitals. However, the consequences on Japanese medical students’ choice of training hospitals varied depending on the region where the university was located and their academic year. The COVID-19 pandemic also influenced the choice of professional interests, even among students who claimed no influence resulted from the pandemic. Notably, a decline was observed in the number of students opting for internal medicine and general medicine compared to pre-pandemic levels, suggesting a potential unconscious bias against departments directly involved in the care of patients during the pandemic. Therefore, further research is warranted to examine how the career paths of students may have changed in the post-COVID-19 era.

### Electronic supplementary material

Below is the link to the electronic supplementary material.

**Supplementary Material 1:** Survey questions

## Data Availability

The datasets used and/or analysed during the current study are available from the corresponding author upon reasonable request.

## Abbreviations

COVID-19: Coronavirus disease 2019
