# Supplementary material for: Influence of the coronavirus disease 2019 pandemic on the post-graduate career paths of medical students: a cross-sectional study
Source: BMC Med Educ. 2024 Jan 10;24:55. doi: 10.1186/s12909-023-05021-6 (PMC10782557; doi:10.1186/s12909-023-05021-6)
Supplement: Supplementary file 1 — Supplementary Material 1: Survey questions [file 12909_2023_5021_MOESM1_ESM.docx]

**Additional File 1. Survey questions**

Q1. What year in medical school are you currently in?

1. MS3
2. MS4
3. MS5
4. MS6

Q2. Which university are you from?

1. Hokkaido University
2. Asahikawa Medical University
3. Sapporo Medical University
4. Hirosaki University
5. Iwate Medical University
6. Akita University
7. Yamagata University
8. Tohoku University
9. Tohoku University of Medical and Pharmaceutical Sciences
10. Fukushima Prefectural Medical University
11. University of Tokyo
12. Tokyo Medical and Dental University
13. Keio University
14. Tokyo Jikeikai Medical University
15. Japan Medical University
16. Tokyo Medical University
17. Nihon University
18. Juntendo University
19. Toho University
20. Teikyo University
21. Showa University
22. Kyorin University
23. Tokyo Women's Medical University
24. National Defense Medical College
25. Saitama Medical University
26. Chiba University
27. Yokohama City University
28. Tokai University
29. Kitasato University
30. St. Marianna University of Medicine
31. Gunma University
32. Jichi Medical University
33. Dokkyo Medical University
34. International University of Health and Welfare
35. Yamanashi University
36. University of Tsukuba
37. Niigata University
38. Kanazawa University
39. Kanazawa Medical University
40. Toyama University
41. Fukui University
42. Hamamatsu Medical University
43. Nagoya University
44. Nagoya City University
45. Aichi Medical University
46. Fujita University of Health Sciences
47. Shinshu University
48. Gifu University
49. Osaka University
50. Osaka City University
51. Osaka Medical University
52. Kansai Medical University
53. Kinki University
54. Kyoto University
55. Kyoto Prefectural Medical University
56. Mie University
57. Shiga Medical University
58. Wakayama Prefectural Medical University
59. Nara Prefectural Medical University
60. Kobe University
61. Hyogo Medical University
62. Okayama University
63. Kawasaki Medical University
64. Hiroshima University
65. Tottori University
66. Shimane University
67. Yamaguchi University
68. Kagawa University
69. Tokushima University
70. Kochi University
71. Ehime University
72. Kyushu University
73. Fukuoka University
74. University of Occupational and Environmental Health
75. Kurume University
76. Nagasaki University
77. Saga University
78. Oita University
79. Kumamoto University
80. Miyazaki University
81. Kagoshima University
82. University of the Ryukyus

Q3. Do you have any concerns about the impact of the COVID-19 pandemic on your future choice of training hospitals or your future career?

1. I am very concerned that it will affect my future career.
2. I think it will affect my choice of initial training hospital, but beyond that, I am not sure.
3. I am not particularly anxious.
4. I do not know.

Q4. Name of the prefecture where your university is located.

[Dropdown list of 47 prefectures of Japan]

Q5. Is the prefecture where your university is located the same as your hometown?

1. Yes
2. No

Q6. Is the prefecture where your university is located the same as the prefecture where your desired training hospital or matched hospital is located?

1. Yes
2. No

Q7. Reason for choosing the region where the training hospital is located.

1. Close to hometown
2. Affiliated to my university hospital
3. There is a medical department that I want to join in the future
4. The program seemed interesting regardless of the location
5. Could not go outside the prefecture where my university is located because of the pandemic
6. Other
7. Have not thought about the future yet (only asked to MS3–5 as it was after MS6 had already completed their match)

Q8. Did the COVID-19 pandemic affect your choice of training hospital?

1. Yes
2. No

Q9a. What are the negative impacts?

1. Difficult to go on an on-site visit
2. Online information sessions are insufficient in conveying the atmosphere of the program/hospital/staff.
3. Participation in away rotations is limited
4. Difficult to prepare for the final interview online (only MS6)
5. The travel restrictions made it more competitive within the prefecture where the university is located (only MS6).
6. Limited opportunity to practice for the interview and matching examination with classmates.
7. Other

Q9b. What are the positive impacts?

1. More time to gather information and consider hospitals that were not on my list of choices.
2. Online information sessions gave more opportunities to appeal to the program staff. (Beneficial for programs that are far away and would have allowed a visit during vacation periods)
3. Other.

Q10. What kind of changes did the COVID-19 pandemic have on the criteria for your training hospital?

Q11. How many hospitals do you plan to visit, or have you visited?

1. 0
2. 1–5
3. 6–10
4. More than 10

Q12. Is the number of hospitals you plan to visit or have visited fewer than you had originally planned?

1. Yes
2. No

Q13. When did you start visiting hospitals, or when do you plan to start?

1. MS3 or earlier
2. Spring between MS3 and MS4
3. Summer of MS4
4. Winter of MS4
5. Spring between MS4 and MS5
6. Summer of MS5
7. Winter of MS5
8. Spring between MS5 and MS6
9. Summer of MS6

Q14. Has the starting time for on-site visits been shifted due to the COVID-19 pandemic?

1. Early
2. Delayed
3. Unaffected

Q15. What are your concerns regarding the preparation for the matching process?

1. Preparation changed depending on whether the interview was online or on-site.
2. Not having adequate extracurricular activities to write on a CV
3. National examination
4. Making connections through on-site visits and away rotations
5. Did not participate in the matching process; therefore, there are no concerns to report (such as university policies)
6. Other.

Q16a. Are you satisfied with the final match? (only MS6)

1. Yes
2. No
3. Did not participate in the matching process

Q16b. If you answered 'no', please tell us why. (only MS6)

Q17. What career do you hope to pursue in the future?

1. Physician
2. Basic researcher
3. Administrative officer
4. Undecided

Q18a. Did the COVID-19 pandemic change the department of your choice?

1. Yes
2. No

Q18b. If you answered "yes", please explain why.

Q19. What is your speciality of interest?

1. Undecided
2. Internal medicine
3. Paediatrics
4. Dermatology
5. Psychiatry
6. Surgery
7. Orthopaedics
8. Obstetrics & Gynaecology
9. Ophthalmology
10. Otorhinolaryngology
11. Urology
12. Neurosurgery
13. Neurology
14. Radiology
15. Anaesthesiology
16. Emergency Medicine
17. Plastic Surgery
18. Rehabilitation Medicine
19. General Medicine
20. Pathology
21. Clinical Laboratory
22. Other

Q20a. Did the COVID-19 pandemic affect the speciality of interest?

Q20b. If you answered 'yes', please explain why.

Q21. Were there opportunities for you to be proactive in extracurricular activities during the pandemic?

1. Increased
2. Decreased
3. Unaffected

Q22. What extracurricular activities are you currently involved in outside of clinical training and lectures at university?

1. Internship
2. Volunteer work
3. Part-time job
4. Leadership (representing an organisation on or off campus)
5. Research activity
6. Connection making
7. On-campus seminars
8. Off-campus seminars
9. Family responsibilities
10. Hobbies
11. Exercise
12. Self-care
13. Preparation for national examinations, OSCE and CBT
14. External Examinations
15. Qualifications
16. Independent study (medical)
17. Independent study (non-medical)
18. Not involved in extracurricular activities due to limited opportunity
19. Other

Q23. Was there any support or information you wanted about the process of choosing a training hospital?
